# Decision-support system for live detection of *Leishmania* parasites from microscopic images with deep learning

**DOI:** 10.1186/s12879-026-13278-7

**Published:** 2026-04-23

**Authors:** Juliane Pfeil, Ahmad Amro, Marcus Frohme, Nils Körber, Daniel Lode, Torben Gloyer, Aisha Gashout, Hamida Al-Dwibe, Alina Nechyporenko

**Affiliations:** 1 Centre for Artificial Intelligence in Public Health Research (ZKI-PH), Robert Koch Institute, Nordufer 20, 13353 Berlin, Germany; 2 Faculty of Pharmacy, Al-Quds University, Jerusalem, Palestine; 3 Division Molecular Biotechnology and Functional Genomics, Technical University of Applied Sciences, 15745 Wildau, Germany; 4 Faculty of Medical Technology/ Medical Laboratory Science, University of Tripoli, Tripoli, Libya; 5 Faculty of Medicine, Dermatology Department, University of Tripoli, Tripoli, Libya; 6 Department of Systems Engineering, Kharkiv National University of Radio Electronics, Kharkiv, 61166 Ukraine

**Keywords:** *Leishmania* parasites, Live/real-time detection, Decision-support system, Deep learning, Object detection, Microscopy

## Abstract

**Background:**

Leishmaniasis is a vector-borne parasitic disease caused by *Leishmania* protozoa. The disease manifests in several clinical presentations including cutaneous, mucocutaneous, and visceral leishmaniasis. The diagnosis of leishmaniasis is complex and often requires a combination of clinical assessment, microscopy, serological tests, and molecular techniques especially in immunocompromised cases. However, traditional diagnostic methods have limitations in terms of accuracy, sensitivity, and the expertise required, leading to an urgent need for advanced, automated diagnostic tools. The aim of this research is to develop a deep learning-based decision-support system for the microscopic examination of tissue samples to support non-experts with the live diagnosis of the disease.

**Methods:**

Tissue samples from lesions were collected from patients diagnosed with cutaneous leishmaniasis in Libya and Palestine for the purpose of preparing microscopic slides. The samples were then visualized using a high-performance laboratory microscope and a mobile, low-cost device. The captured images were subsequently used to train the object detection framework YOLOv8 with the aim of identifying *Leishmania* parasites. A graphical user interface was developed for the application of the deep learning model, which enables real-time detection of the parasites using a microscope camera, as well as recognition from previously generated images and videos.

**Results:**

The deep learning YOLOv8 framework was successfully trained using data generated by the advanced microscope and employed for the detection of *Leishmania* parasites. Subsequent finetuning with a combined set containing the aforementioned data and microscopic images generated with the low-cost device resulted in a considerable improvement in accuracy. The efficacy of the model was demonstrated through its successful operation on previously unseen data. Object detection yielded a mean average precision of 0.78 for the combined datasets. The evaluation process for determining the presence of parasites in an image resulted in 91% accuracy, 91% sensitivity, 90% specificity and 94% precision on the test data.

**Conclusions:**

Deep learning-based YOLOv8 achieved accurate *Leishmania* detection in tissue samples, enhancing decision-support for non-experts via real-time graphical user interface support. This innovation can simplify diagnostics by addressing traditional method limitations, enabling early, accessible leishmaniasis detection in resource-limited settings, and potentially inspiring similar applications in other parasitic diseases.

**Clinical trial number:**

Not applicable.

## Background

Leishmaniasis is a significant public health problem in tropical and subtropical regions, affecting an estimated 12 million people worldwide, with approximately 1.5 to 2 million new cases reported annually [1]. The World Health Organization (WHO) has classified leishmaniasis as one of the neglected tropical diseases (NTDs), primarily affecting impoverished populations with limited access to healthcare services. The disease burden is further exacerbated by the underreporting of leishmaniasis, due to the challenges of diagnosis and the associated stigma of visible lesions [2, 3].

The clinical manifestations of leishmaniasis are diverse and depend on the *Leishmania* species, the host’s immune response, and the geographical region. Cutaneous leishmaniasis (CL) is the most common form, characterized by ulcerative skin lesions that may heal spontaneously but can leave disfiguring scars. Mucocutaneous leishmaniasis (MCL), primarily caused by *Leishmania braziliensis*, leads to the destruction of mucous membranes of the nose, mouth, and throat, often resulting in severe deformities. Visceral leishmaniasis (VL), also known as kala-azar, is the most severe form, caused by species such as *L. donovani* and *L. infantum*. VL affects internal organs like the spleen, liver, and bone marrow, and if left untreated, it can be fatal [4].

Accurate diagnosis of Leishmaniasis is crucial for the effective management and treatment. Traditional diagnostic methods include direct microscopic examination of Giemsa stained smears, cultures, and serological tests. Molecular approaches including polymerase chain reaction (PCR) have also been developed to detect parasite DNA with high sensitivity. However, these methods are not without limitations. Microscopy, while cost-effective, is labor-intensive and requires skilled personnel. Serological tests can lack specificity and sensitivity. Molecular methods, although highly sensitive, are expensive and require specialized equipment and expertise, limiting their accessibility in resource-poor settings [5]. Given these obstacles, there is a persistent need for the development of novel diagnostic tools that are accurate, cost-effective, and accessible.

In recent years, advances in artificial intelligence (AI) and machine learning (ML)/deep learning (DL) have shown great potential in the field of medical diagnostics. AI-driven approaches can automate the analysis of microscopic images, enhancing the accuracy and speed of diagnosis while reducing the reliance on highly trained personnel [6]. Several studies have explored the use of DL in the diagnosis of parasitic infections, including malaria, toxoplasmosis, and Chagas disease. These investigations have demonstrated the potential of AI-driven methods to outperform traditional diagnostic techniques in terms of accuracy and speed [7].

However, the application of DL to microscopic leishmaniasis diagnosis remains underexplored. Most existing studies on leishmaniasis detection focus on traditional image analysis methods [8–12], with limited research on AI-based approaches. Furthermore, the few studies that have investigated ML/DL for leishmaniasis detection [13–23] have been limited by small datasets, lack of external validation, and insufficient focus on the diverse clinical presentations of the disease [24, 25].

This represents a significant gap in the literature, highlighting the need for robust, well-validated AI models that can accurately detect and classify *Leishmania* parasites. While some studies have been published on the use of ML/DL models for tasks such as object detection, segmentation, and classification, there remains a lack of applications that support decision-making in diagnosing *Leishmania*. Existing literature highlights the availability of open-source software designed for the automated quantification of *Leishmania* for research purposes, along with a so-called reconfigurable autonomous microscopy platform [26]. This platform is equipped to perform automated slide scanning and provides both brightfield (BF) and fluorescence imaging capabilities, which can reduce the cost of tedious work and human error. It is asserted by the authors that the detection of *Leishmania* should be considered as a potential application of this technology, however, no verification of its feasibility has been carried out.

However, in regions with poor socioeconomic conditions, high population mobility, and complex environmental and climatic changes, prevention and control of the spread of leishmaniasis are of great importance. In addition, there is a particular shortage of qualified medical personnel, so decision support systems (DSS) for early diagnosis based on artificial intelligence can significantly shorten the diagnosis time and thus reduce the morbidity and mortality rate.

The aim of this study is to provide a DSS for the point-of-care detection of leishmaniasis. The development and evaluation of a DL-based framework for the live detection of *Leishmania* parasites from microscopic BF images will serve as the foundation for this endeavor. The proposed model will be trained on a large and diverse dataset, comprising skin samples from lesions with CL, ensuring its applicability across different microscopic and clinical settings.

## Methods

### Study design

Developing a DSS for real-time recognition of *Leishmania* parasites requires a multidisciplinary effort, integrating DL, software engineering, and domain-specific knowledge. Collaboration with medical professionals is crucial to ensure the system’s effectiveness and accuracy in a clinical setting. Study design involves several steps, from data collection to model deployment and user interaction:

1. **Dataset collection and image labeling**
  A total of 734 BF microscopic images from approximately 250 patients with CL from Libya and Palestine were collected in two iterations to create a diverse dataset. The images were then subjected to a rigorous evaluation by experts, who distinguished between positive (i.e., *Leishmania* amastigotes present) and negative samples. The individual parasites were also labelled with bounding boxes.
2. **DL framework development, evaluation and optimization**
  The DL object detection framework YOLOv8 [27] was selected for parasite identification due to its capacity for accurate and real-time prediction. The performance of the selected DL model is evaluated in terms of detection accuracy, and an optimization of the hyperparameters is conducted.
3. **Graphical user interface (GUI) design**
  A GUI is being developed to facilitate the delivery of the DSS. This interface will enable the user to perform direct image analysis during BF microscopy using a camera or to load recorded pictures and videos from memory.

### Dataset collection and image labelling

A total of 244 Libyan patients with CL clinically diagnosed by PCR at Tripoli University Hospital were studied to establish a representative dataset. PCR confirmation corresponded to the same lesion from which samples were obtained by scraping of the affected skin (slit-skin smears or touch smears). The collected material was applied to glass slides, air dried, fixed with methanol, and staining with Giemsa was conducted. This enhanced the visibility of *Leishmania* amastigotes bodies, which were present in the samples. A digital microscope (Keyence BZ9000E) was used in a laboratory environment to capture multiple images of each slide using an oil immersion objective (numerical aperture (NA) 1.3, resolution 0.21 μm) with 100x magnification. The resulting dataset comprises 570 high-resolution images. Of these, 350 are positive images, representing a range of parasite densities (1-100 *Leishmania* bodies per image). Additionally, 220 negative images were obtained without parasites, serving as a control. This dataset was used for the initial training of the DL algorithms for parasite detection.

To provide a robust DSS that is both mobile and cost-effective, and to demonstrate its applicability to new data in resource-constrained environments, an additional dataset was generated with six patients from Palestine. For this approach the Bresser Erudit DLX microscope [28], in conjunction with the Bresser MikroCam SP 5.0 camera [29], was employed for the analysis of additional laboratory slides. The microscope is designed to be portable and is powered by a rechargeable battery. The resulting dataset comprises 106 positive and 58 negative images, recorded with an oil immersion objective (NA 1.25, resolution 0.22 μm) with 100x magnification.

The images from both microscopes were scaled to a size of 1920 × 1440 pixels (px), which corresponds to the standard output format of the Keyence microscope.

**Fig. 1:**
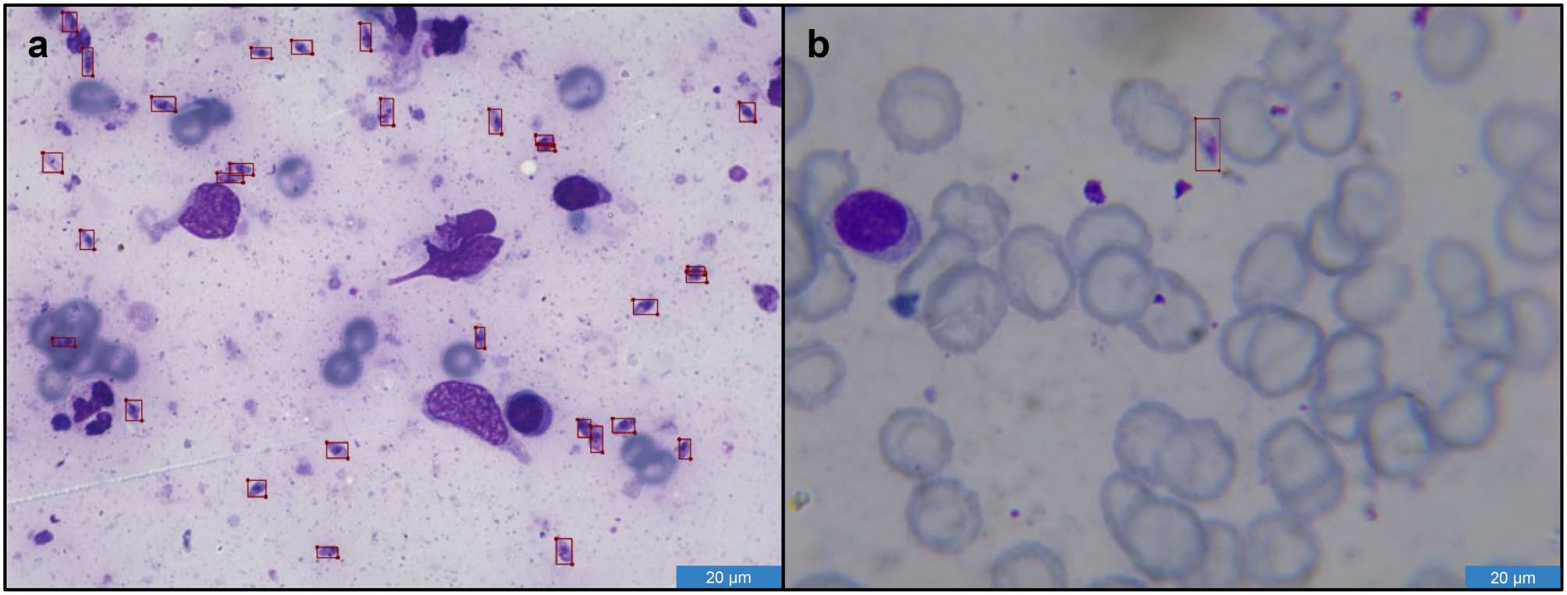
Microscopic images of *Leishmania* parasites at 100x magnification. *Leishmania* bodys are labeled with red boxes from the software LabelMe [30]. **a** Image taken with the microscope Keyence BZ9000E. **b** Image taken with the microscope Bresser Erudit DLX

To ensure precise image labeling, specifically identifying the presence of *Leishmania* parasites, close collaboration with medical specialists is essential. The labeling was done using the software “LabelMe” [30] by experts, who marked the regions affected by *Leishmania* amastigotes using bounding boxes. Annotations followed a sequential expert review with consensus resolution of uncertain cases to ensure quality control. Both individual *Leishmania* and groups were classified as “parasites”. In densely packed regions, overlapping amastigotes were sometimes annotated with a single box because reliable instance-level delineation was not consistently possible; this pragmatic strategy reduces ambiguity and supports robust training labels. Non-parasitic structures visible in Giemsa-stained smears (e. g., stain precipitates, debris, and other artifacts) were not annotated and were therefore implicitly treated as background during training. Figure 1 shows two images with high and low parasite density taken with the Keyence BZ9000E and the Bresser Erudit DLX:

The dataset that was created with the Keyence BZ9000E and the Bresser Erudit DLX are described in Table 1:

**Table 1:** Microscopic *Leishmania* datasets

Dataset	Microscope	# Patients	# Images		# Leishmania parasites
			Positive	Negative	
dataset 1	Keyence BZ9000E	244	350	220	2,224
dataset 2	Bresser Erudit DLX	6	106	58	957

The datasets were partitioned into 70% training, 15% validation, and 15% test sets ensuring a slide-wise split, each set containing 61–65% positive and 35–39% negative images.

To quantitatively assess annotation consistency, an inter-annotator agreement analysis was performed on a randomly selected subset of 50 images from dataset 1 and 2. Two experts independently labeled the images blinded to each other’s results. Bounding boxes were matched using an interception over union (IoU) threshold of ≥ 0.5. Agreement was assessed using mean IoU and mean average precision (mAP). In addition, an image-level agreement was evaluated by classifying each image as positive (≥ 1 parasite) or negative. Inter-annotator consistency was quantified using Cohen’s kappa (κ), providing a chance-corrected measure defined as:

$$\:\kappa\:=\frac{{P}_{o}-{P}_{e}}{1-{P}_{e}}$$

where P_o_ represents the measured proportion of agreement and P_e_ the random expected agreement based on marginal probabilities.

### DL framework development, evaluation and optimization

The YOLOv8 model [27] was selected for object detection due to its capacity to deliver rapid, precise, and real-time predictions within a unified end-to-end network architecture, thereby optimizing efficiency for both research and deployment contexts. YOLO (You Only Look Once) is a convolutional neural network that predicts bounding boxes and class probabilities of an image in a single forward pass. YOLOv8 employs the sigmoid linear unit (SiLU) activation function, a development that has facilitated enhancements in gradient flow and feature expressiveness. It shows excellent results and is one of the most popular and effective models for real-time object detection and image segmentation.

The standard YOLOv8n (smallest model of this series, pre-trained on the common object in context (COCO) dataset [31]) architecture was chosen to enable practical inference in low-resource settings. The model was trained using the Weights & Biases platform [32], facilitating the optimization of experimental monitoring, the validation of model checkpoints, and the graphical representation of model performance, thereby completing the hyperparameter tuning procedure, with the following ranges being evaluated:

- epochs 30–150.
- batch size 6–64.
- learning rate 0.0001-0.05.

The main objective was to maximize the mAP at an IoU of 0.5.

$$\:mAP\:=\frac{1}{N}\sum\limits_{i=1}^{N}A{P}_{i}$$

mAP is the mean of the average precision (AP) of different classes (N) in a dataset. This considers both precision (number of correctly detected objects) and recall (number of actually detected objects). For model training the learning rate and batch size were adapted for the existing graphics processing unit (GPU) infrastructure (GPU NVIDIA Tesla V100, DDR4-RAM 384 GB). The hyperparameters that yielded optimal performance are as follows: epochs = 120, learning rate = 0.0001, and batch size = 6. The training of the neural network was continued until the mentioned number of epochs, at which point no additional significant loss could be identified.

Dataset 1 was utilized to train a ‘laboratory model’, given that the images contained therein had been captured using the advanced Keyence microscope. A second model was finetuned based on the above model using a combination of datasets 1 and 2. Due to the more heterogeneous nature of the data, this model was designated as ‘universal’.

The final, optimized models were employed to evaluate the mAP for predicting Leishmania parasites and assessing the accuracy of the detection boxes on independent test sets. Furthermore, the accuracy, sensitivity, specificity and precision for the detection of positive (parasite present) and negative (no parasite present) images were calculated with the true positives (TP), false positives (FP), true negatives (TN), and false negatives (FN). An image was classified as parasite-positive if it contained at least one predicted bounding box with a confidence score ≥ 0.25 and an IoU ≥ 0.5 relative to a ground-truth annotation. Non-maximum suppression (NMS) used default YOLOv8n parameters (IoU NMS threshold = 0.7). The following equations were utilized for the calculation:

$$\:accuracy\:=\frac{TP+TN}{TP+FP+FN+TN}$$

$$\:sensitivity=\frac{TP}{TP+FN}$$

$$\:specificity=\frac{TN}{TN+FP}$$

$$\:precision=\frac{TP}{TP+FP}$$

TP, TN, FP and FN represent the predictions for the validation and test datasets. The Wilson score method for binomial proportions was used to calculate the 95% confidence intervals (CI) for performance metrics.

### Graphical user interface design

Integration of the trained model into a system for live analysis involves developing a software interface that can interact with microscopic hardware. Real-time processing requires the system to be able to pre-process and transfer live images from the microscope to the model, and display the results instantly. To enable interaction between the system and the user, a GUI is designed. It includes a dashboard for displaying microscopic images, uploading pictures/videos and viewing results. The GUI main functions comprise displaying the live image from the microscope camera, taking pictures and selecting a DL model for real time prediction of *Leishmania* parasites.

**Fig. 2:**
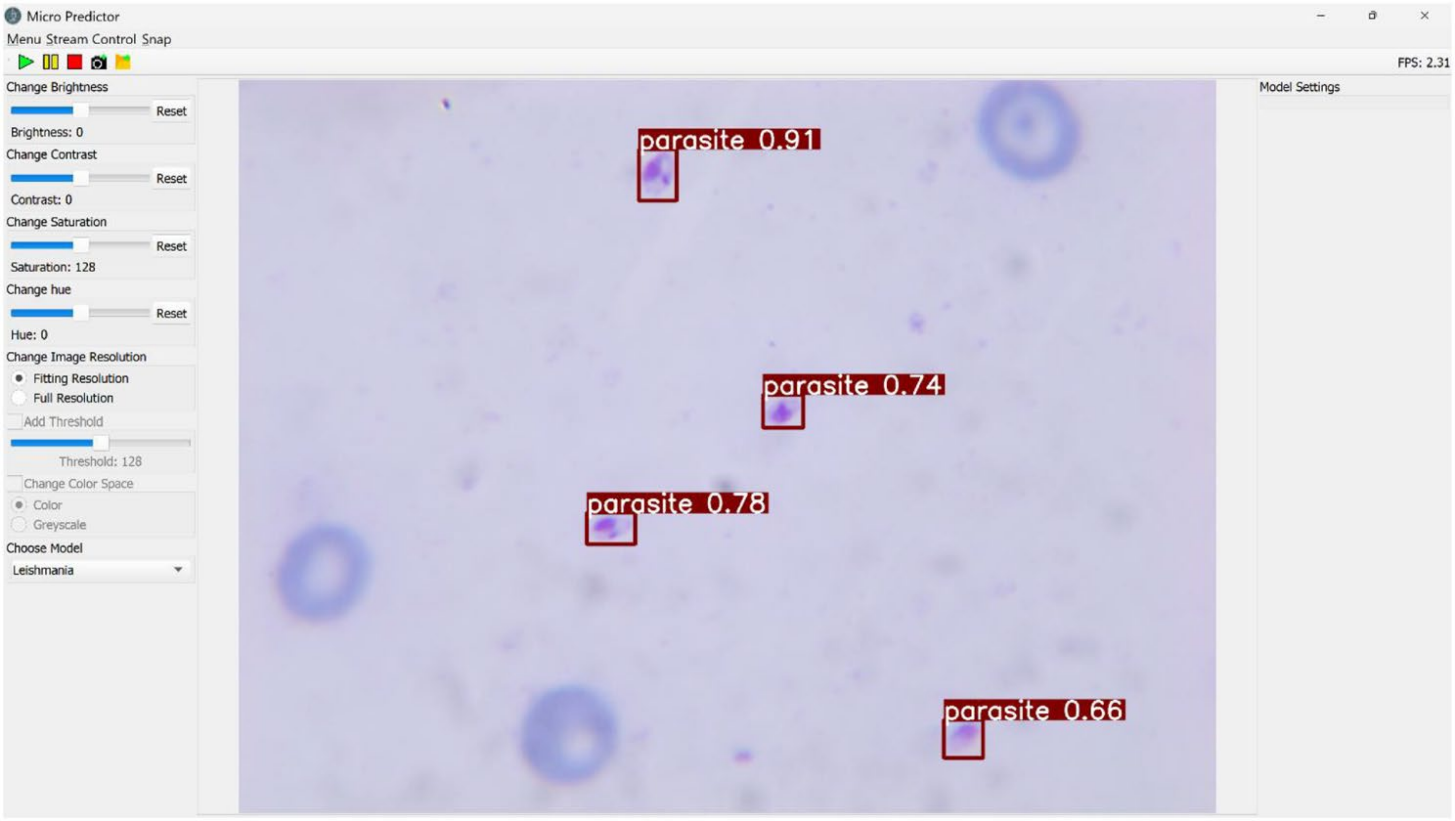
GUI of the DSS for *Leishmania* detection. *Leishmania* parasites are detected by the universal model and marked with red boxes indicating the model’s confidence value

The software is composed of multiple components (see Fig. 2) that execute distinct tasks and functions: In the upper area, image capture can be initiated, storage paths can be designated, and the program can be terminated. The left menu bar contains functions for adjusting brightness, contrast, saturation and hue, changing resolution, manipulating threshold values and altering color space. Furthermore, the model for *Leishmania* detection can be selected or other models for image recognition can be integrated. The live image, accompanied by real-time detections, is displayed in the middle section. The detected parasites along with the confidence value of the model are marked with red boxes.

The GUI is compatible with Windows and Mac operating systems and was tested with the following specifications: Windows 10, 32GB RAM, CPU: Intel Core i9-9820X, GPU: 2x NVIDIA GeForce RTX 2080Ti and MacOS Sonoma,16GB RAM, CPU: Apple M1.

This setup enables both healthcare professionals and non-specialists to easily operate the system and display the results.

## Results

### Model evaluation

This section presents the results of object detection using YOLOv8 for the identification of *Leishmania* parasites. One model was trained using dataset 1, which contained only images captured using the state-of-the-art laboratory microscope. This model is therefore referred to as the ‘laboratory model’. An additional model was finetuned based on the laboratory model using a combination of datasets 1 and 2. Dataset 2 was created with the mobile microscope, and the resulting model trained with both datasets is therefore referred to as the ‘universal model’.

**Fig. 3:**
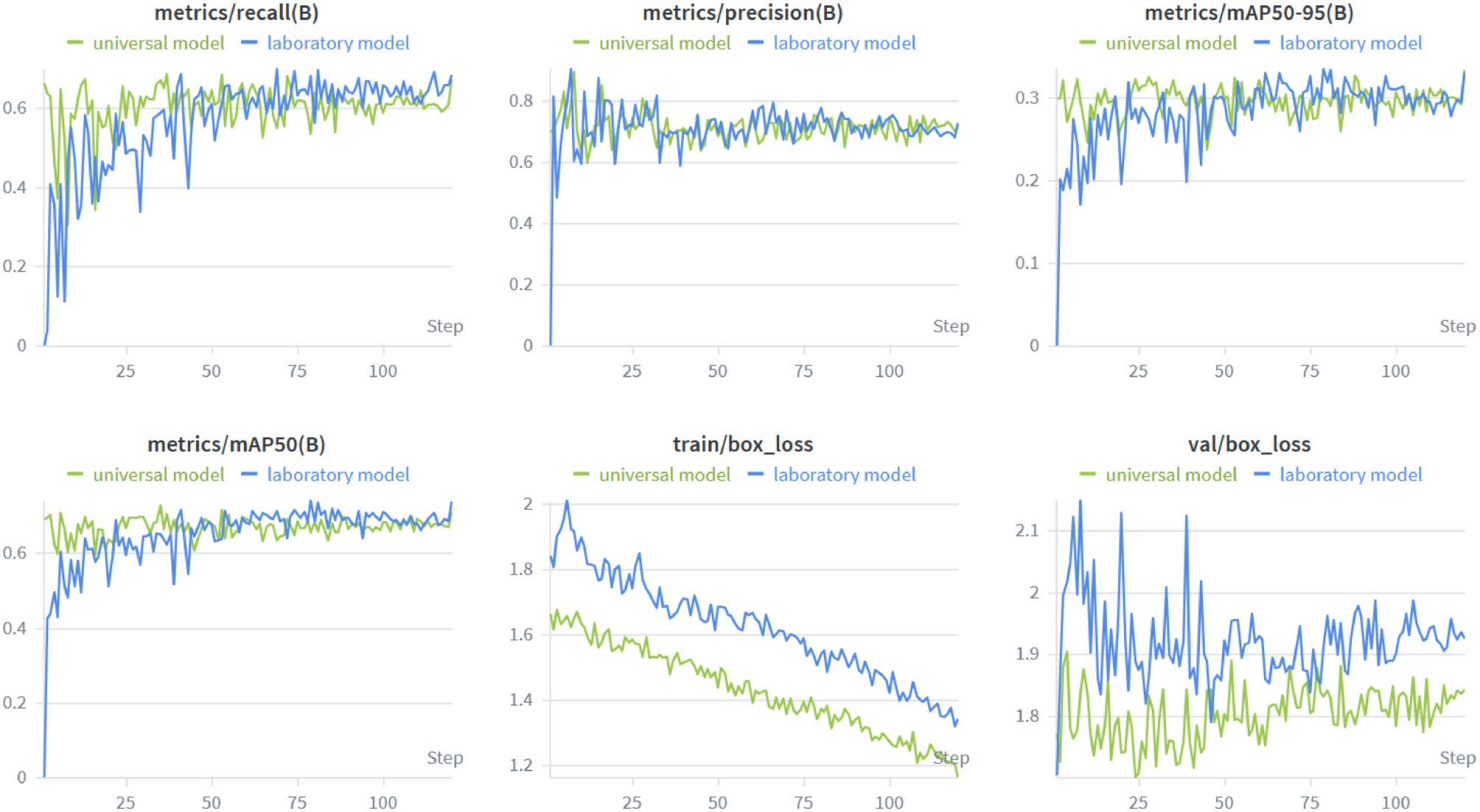
Training and validation metrics for the laboratory and the universal model. The metrics recall, precision, mAP at an IoU between 0.5 and 0.95 and at 0.5 (B stands for box) are shown, as well as the box loss for the training and validation data

The training and validation performance of both models was evaluated over 120 epochs. The results are shown in Fig. 3, which tracks the progression of the box loss and performance metrics. The models’ detection performance, measured using precision, recall, and mAP, showed continuous improvement over time. Notably, a comparison between the two models reveals several key differences:

The laboratory model consistently achieved higher performance metrics, particularly in precision, recall, mAP50, and mAP50-95. For example, mAP50-95 increased steadily and reached a maximum of 0.34 for the laboratory model, while the universal model reached approximately 0.33. Thus, both models demonstrate good object detection ability, which is likely applicable in practical scenarios.

Similarly, the laboratory model maintained slightly higher precision and recall values throughout training. This suggests that the laboratory model is better optimized for the dataset it was trained on.

On the other hand, the universal model achieved lower training box loss, indicating a better fit to its training data. However, it exhibited higher and more volatile validation box loss, indicating potential challenges in generalization due to the increased complexity of the data introduced by mobile microscope images.

Despite these differences, both models exhibited no significant signs of overfitting, and the universal model demonstrate strong performance overall, especially considering its broader intended application across varied imaging conditions of datasets 1 and 2. These results underscore the trade-off between generalizability and task-specific optimization, with the universal model offering improved tolerance to variability between the two acquisition setups used in this study, and the laboratory model excelling within its original setting.

Finally, the mAP was calculated at an IoU of 0.5 on the test data from dataset 1 + 2. The laboratory- and the universal model achieved a value of 0.78.

### Qualitative detection results

**Fig. 4:**
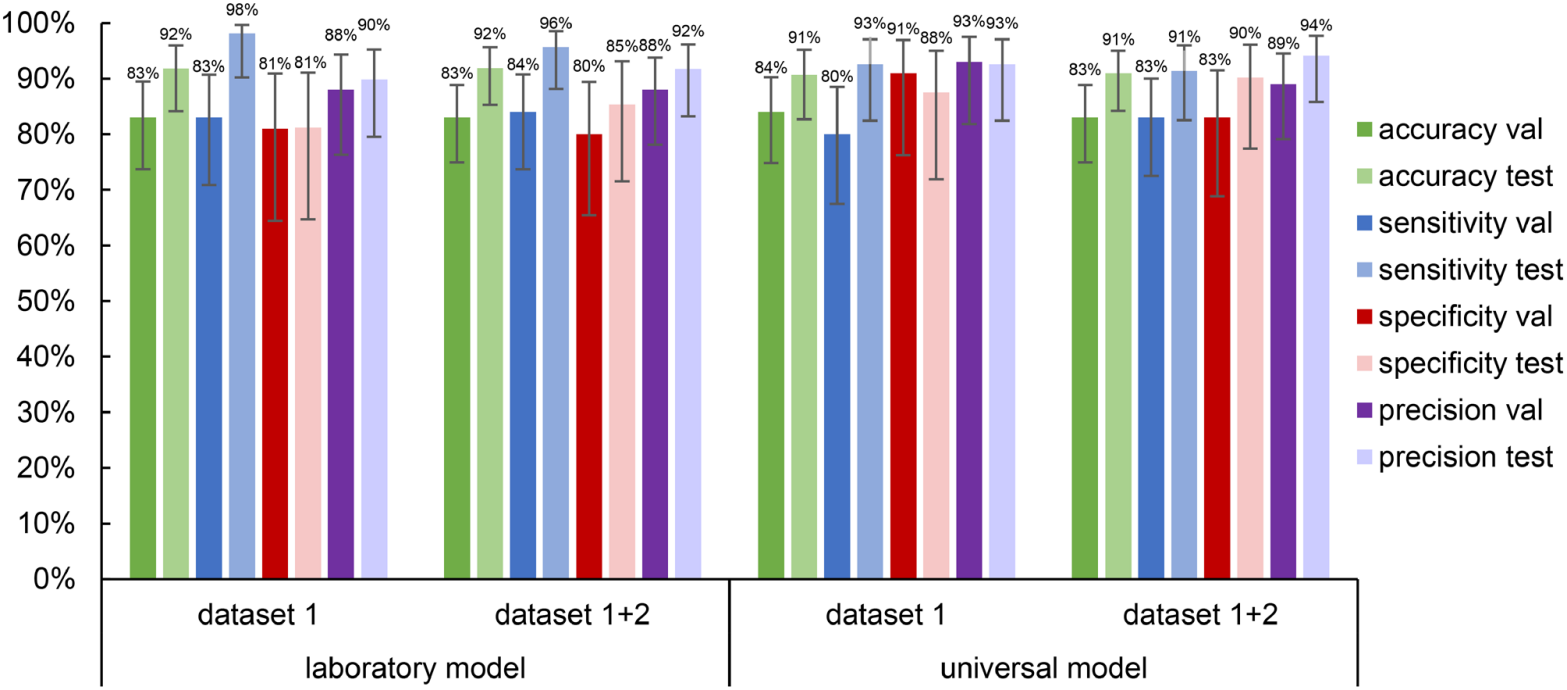
Accuracy, sensitivity, specificity and precision of the laboratory and universal model on dataset 1 and 2. The metrics were calculated based on the images that were detected as positive or negative (with or without parasites) from validation and test sets. Error bars indicate 95% CI calculated using the Wilson score method for binomial proportions. The laboratory model was trained on dataset 1, and the universal model on datasets 1 and 2

In order to evaluate the performance of the models for the targeted DSS, the metrics accuracy, sensitivity, specificity and precision were calculated to differentiate between positive (parasite present) and negative (no parasite present) images. As illustrated in Fig. 4, the values for the laboratory (trained on dataset 1) and the universal model (trained on dataset 1 and 2) are presented for the validation and test data.

The deviations between the validation data and test data are on average 7% for all metrics, indicating stable performance within the distribution of the collected datasets. The laboratory model achieves an accuracy of 92% on the test data from dataset 1 and the combined datasets. The sensitivity, specificity and precision metrics also demonstrate comparable values between the two datasets. This finding indicates that the high-quality dataset 1, which was recorded with the advanced microscope, already provided a suitable foundation for the analysis of new data recorded with lower resolution using the mobile microscope.

Despite this, the potential benefits of finetuning the laboratory model with the combined dataset should be assessed to ascertain whether further enhancements in performance could be achieved. The findings derived from the universal model indicate an improvement, both in the combined dataset and in the original dataset 1. The evaluation of the test data demonstrated an accuracy of 91%, with a sensitivity average of 92% and a specificity that exhibits an enhancement of up to 7%. The precision achieves an average value of 92% for both datasets, indicating a low proportion of FP detections related to staining artefacts or debris. Specifically, for the validation data the model demonstrates an average enhancement of 2% across all metrics. Despite the fact that the improvements are not excessive, it may nevertheless be beneficial to finetune the model with novel data and diverse microscopes.

Furthermore, the performance of the universal model was evaluated using images with low and high *Leishmania* densities to assess the diagnostic challenges posed by low parasitemia. Parasite density was defined using the 25th and 75th percentiles of parasite counts across the combined dataset 1 + 2 distribution (low: =1 parasite/image; high: ≥5 parasites/image). On the test set, the universal model achieved 92% accuracy on low-density images and 100% accuracy on high-density images, indicating reliable performance across *Leishmania* densities with only a modest decrease at low parasitemia.

In order to evaluate the process of the dataset labelling, an inter-annotator agreement examination was performed on a subset of 50 images. Object level agreement demonstrated high concordance with a mean IoU of 0.69 and a mAP of 0.8. At the image level (positive vs. negative classification), agreement between annotators yielded a Cohen’s kappa of κ = 0.92, indicating “almost perfect” agreement. The findings of this study suggest that the annotations made by experts are consistent, thereby providing a reliable foundation for the ground truth labels.

### Performance with unseen data

**Fig. 5:**
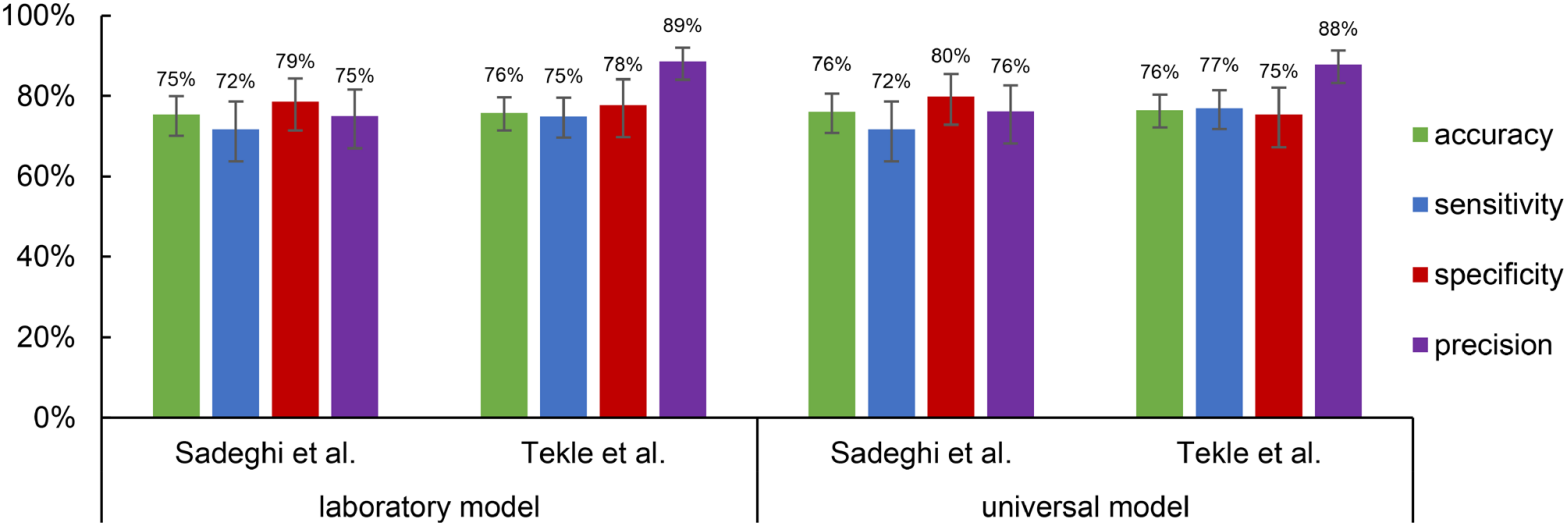
Accuracy, sensitivity, specificity and precision of the laboratory and the universal model with unseen data. The metrics were calculated based on the images that were detected as positive or negative (with or without parasites) from datasets [33] and [34]. Error bars indicate 95% CI calculated using the Wilson score method for binomial proportions

In order to thoroughly test the performance and generalizability of the models, they were applied to previously unseen data. For this purpose, the datasets from Sadeghi et al. [20, 33] and Tekle et al. [21, 34] were downloaded. Dataset [33] contains a total of 292 images from CL patients, of which 138 are classified as positive and 154 as negative. Dataset [34] contains a total of 417 images including 247 with CL, 44 with VL and 126 negative. In both datasets, the samples were stained with Giemsa and images were captured at 100x magnification. Utilising the aforementioned data, the analysis of images captured with various microscopes, under different parameters (exposure, contrast, etc.) and that exhibit indications of the disease (CL/VL) can be conducted. The laboratory and universal model were applied directly to these images without any preprocessing in order to test their capabilities in real-world conditions. Performance metrics are shown in Fig. 5.

The laboratory model achieves accuracies of 75% and 76% for both datasets. Analogous values are attained for sensitivity, specificity and precision. The universal model demonstrates an improvement of up to 2% over the laboratory model in both datasets. The enhanced performance of the universal model is apparent, as it has been trained with data from two distinct microscopes at disparate facilities. While these results are lower than those reported by models trained directly on the respective public datasets, this difference is expected and is consistent with domain shift and dataset-specific optimization. It can be hypothesized that further finetuning with novel datasets would enhance performance. Nevertheless, even when applied in a completely different setup, the models maintain moderate performance under external domain shift without additional finetuning. Overall, our YOLOv8-based universal model demonstrates considerable promise because it provides direct, object-level localization of amastigotes and remains effective across both laboratory and portable image acquisition in the conducted study.

## Discussion

A variety of research projects employ special staining techniques to identify *Leishmania* parasites using fluorescence microscopy (FM) [8, 9, 11, 26]. Although the technique is highly sensitive, it requires the use of specific fluorescent dyes and advanced microscopes for visualization.

Ouertani et al. [8, 11] employed FM to investigate the automatic segmentation of *Leishmania* parasites on indirect immunofluorescence images, using both classical, such as Otsu thresholding or the watershed algorithm and ML-based (K-means clustering) segmentation methods. These approaches were tested on 40 images containing around 1,500 parasites, achieving a correct segmentation rate of between 70% and 85%. While the method demonstrates potential, its performance on overlapping objects, its reliance on specific imaging conditions and manual thresholds, and its use of a single unsupervised ML approach on a limited dataset suggest opportunities for enhancing its generalizability. In [9] the researchers presents a robust and adaptable software tool for automated parasite quantification in microscopy images, primarily stained with DAPI. It enhances throughput and consistency in parasite research and screening. Despite INsPECT [9] remains a practical and valuable tool, particularly for labs without access to advanced computational resources or labeled training data, the integration of ML or data-driven models could enhance adaptability to variations in staining, imaging conditions, and parasite morphology. A research paper [26] presents Octopi, a low-cost, modular, and autonomous microscopy platform designed for scalable and automated diagnostics with potential applications for *Leishmania* detection. Octopi combines automated slide scanning, real-time computer vision, and ML-based classification, achieving high throughput and diagnostic accuracy. Although currently validated only for malaria, extending the platform to *Leishmania* detection and optimizing spectral staining compatibility present clear directions for future development.

A subset of scientists is also focusing on the diagnosis of the visceral form, a process that requires the collection of bone marrow samples [10, 12, 17, 18]. Given its invasive nature and dependence on skilled, precise processing, there is a clear necessity to develop less intrusive and more streamlined approaches.

In study [10] a method for segmenting *Leishmania* bodies in microscopic images using contrast stretching, morphological processing and a modified Chan-Vese level set algorithm was developed. While currently constrained by the small dataset (28 parasites) and a global error of ~ 11%, expanding the dataset and refining the model could substantially improve applicability. Isaza-Jaimes et al. [12] propose a computational method for automatically detecting parasites in bone marrow images. Their approach uses image preprocessing to enhance edges, followed by region selection based on intensity profiles and shape-based analysis for classification. The conventional image processing approach was unsuccessful in identifying 20% of parasites in 45 images. However, the integration of more advanced ML/DL techniques could enhance the performance of parasite detection. Gonçalves et al. [17, 18] focuses on developing an automatic diagnostic system for VL using DL classification and segmentation models trained on bone marrow microscopy images. While the proposed computer vision-based approaches achieved ~ 99% accuracy, expanding beyond the small dataset (150 images) and conducting real-world validation would be essential to strengthen generalizability and clinical applicability.

Given the focus of the present research work on the development of a DSS for the detection of CL from microscopic BF images, it is important to note that there are a number of related studies [13, 14, 19–22] in this context:

In [13] the researchers propose a DL-based solution using a U-Net [35] architecture to automatically segment and classify parasites into promastigotes, amastigotes, and adhered forms. This approach aims to reduce subjectivity and improve diagnostic efficiency by enabling robust detection. Although developed on 45 cell culture images, extending evaluation to larger, real-world datasets would be critical for establishing performance and applicability. Zare et al. [14] presents a ML-based system for the detection of CL in microscopic images using the Viola-Jones object detection algorithm enhanced with AdaBoost. The system achieved a combined sensitivity of 83% and specificity of 35% by detecting both infected macrophages and free amastigotes, offering a rapid and cost-effective diagnostic aid. Although Haar-like features and boosting offer computational efficiency, enhancing accuracy and specificity in individual parasite detection represents a key area for improvement. In [19] a range of DL image classification networks are utilized for the purpose of evaluating microscopic images as either positive or negative. The authors achieve a commendable level of accuracy and specificity, with a maximum recorded value of 99%. Sadeghi et al. [20] also examined various DL image classification networks and the outcomes are comparable, with a sensitivity of 100%, exhibiting a precision and specificity of 98%. The underlying dataset consists of 292 images (138 positive and 154 negative) and is available to download online. Although both approaches are effective overall, the methods could be improved by enabling direct parasite localization. Additionally, the generalizability and high performance of the models in [19] and [20] should be evaluated using more diverse data from other sources. In [21] the researchers investigated a dataset with CL and VL samples and used different object detection models to localize and classify the parasites. YOLOv5 [36] performed best and achieved a mAP (IoU 0.5) of 0.73. The most recent research [22] in this area focuses on the detection of *Leishmania* parasites using advanced image preprocessing techniques in combination with conventional ML algorithms. The proposed approach demonstrates a high level of sensitivity and specificity for the presented dataset, thereby establishing itself as a bridge between the explainable classical methods and the advanced DL models.

The DSS developed in our approach leverages DL-based object detection to classify microscopic BF images with CL while directly localizing *Leishmania* parasites, and it remains applicable across both laboratory and portable image acquisition. It achieves a creditable level of performance presenting a mAP of 0.78, along with 91% (95% CI: 84–95%) accuracy, 91% (95% CI: 83–96%) sensitivity, 90% (95% CI: 77–96%) specificity and 94% (95% CI: 86–98%) precision on the test set and is easy to use via the provided GUI.

However, it is important to note that the study has certain constraints that should be taken into perspective for future applications. The recorded datasets originate from a small number of geographic sites and the size of the cohorts is imbalanced, which may reduce the diversity of biological and epidemiological patterns captured during training and evaluation. In addition, the study is restricted to one specific clinical presentation (CL) and does not encompass the full range of *Leishmania* species and disease forms observed globally. Furthermore, although cases were PCR-confirmed clinically, the model was evaluated only on BF microscopy images; direct, paired benchmarking against more sensitive modalities (e.g., FM or PCR at the specimen level) was not possible with the available paired data. In addition, external validation was limited to the available sites and should be expanded in future prospective, multi-center studies to better assess generalizability across geographies, species, and acquisition conditions, while also incorporating expert human reader assessments on the same specimens to contextualize model performance. To address annotation consistency, a subset-based inter-annotator agreement analysis demonstrated high concordance between experts, supporting the reliability of the ground-truth labels.

Published studies indicate that routine smear microscopy demonstrates variable sensitivity, typically ranging from approximately 50–88% depending on parasite burden and evaluator expertise, with PCR generally showing higher sensitivity of 90–100% [5, 37, 38]. Within this context, our model achieved 91% sensitivity on the internal test set, and maintained external performance of 72–77% sensitivity under domain shift. These results place the system’s performance within the variability observed for human microscopy and support its intended role as a DSS rather than a substitute for expert evaluation. A systematic benchmark against alternative object detection and instance segmentation architectures would also be valuable future work to better characterize performance trade-offs for this task. Nevertheless, our approach already demonstrates satisfactory performance on independent external data from Sadeghi et al. [20] and Tekle et al. [21], representing different geographical sites (Iran, Ethiopia) and clinical manifestations (CL, VL), thereby introducing relevant distributional differences. When applied without site-specific finetuning, the external accuracy reached 76%, the sensitivity ranged between 72 and 77%, and the specificity between 75 and 80%. These results indicate moderate but reduced performance under domain shift. Consequently, performance in new clinical environments should be anticipated within this range, unless local adaptation is undertaken. In order to facilitate broader applicability, the complete codebase and training pipeline are released as open source. This enables site-specific adaptation and finetuning when additional local training data are available from other regions, species and imaging setups. In practice, this process would require a small annotated dataset from the new setting (e.g., 100–200 representative images across positive and negative cases). The lightweight YOLOv8n framework facilitates the rapid finetuning of the model on a single GPU, a process that can be completed in less than an hour (~ 30 min on four GPU NVIDIA Tesla V100). This approach does not necessitate architectural modifications, as the open-source pipeline facilitates incremental retraining with new labelled data.

## Conclusions

In conclusion, our study presents a valuable advancement in the field of automated *Leishmania* parasite detection by leveraging a modern DL-based object detection approach, using YOLOv8 on BF microscopic images. Unlike prior works that either relied on conventional image processing techniques or focused on FM, our method eliminates the need for specialized staining or expensive imaging equipment, which may lower practical barriers in settings where such infrastructure is unavailable. The model directly detects individual parasites, and, within the evaluated cohorts and imaging conditions, demonstrates a valuable level of performance. To support reproducibility and extension, the datasets, labels and models are made available online, together with step-by-step instructions and an easy-to-use GUI-based DSS, enabling straightforward evaluation and finetuning on new data. While this study is focused on CL and the available data are limited in geographic and species diversity, the open-source pipeline facilitates adaptation to additional sites, imaging setups, and *Leishmania* species as future data become available. In addition to its diagnostic capabilities, the developed system may also be useful as a valuable educational tool. By providing precise and real-time visual localization of parasites, it can assist in training clinicians, laboratory technicians, and medical students to better recognize parasitic forms and improve their diagnostic skills. This makes the system not only a useful tool in clinical settings but also a practical solution for capacity building and education in endemic regions.

## Data Availability

The datasets generated and analyzed during the current study are available at Zenodo: (10.5281/zenodo.17964511). Besides, the developed algorithms, trained models and detailed installation instructions for the DSS are available at: (https://github.com/ZKI-PH-ImageAnalysis/leishmania).

## Abbreviations

AI: Artificial intelligence
AP: Average precision
BF: Brightfield
CI: Confidence interval
CL: Cutaneous leishmaniasis
COCO: Common objects in context
DSS: Decision support system
FM: Fluorescence microscopy
FN: False negative
FP: False positive
GPU: Graphics processing unit
GUI: Graphical user interface
IoU: Interception over Union
mAP: Mean average precision
MCL: Mucocutaneous leishmaniasis
NMS: Non-maximum suppression
DL: Deep learning
NA: Numerical aperture
NTD: Neglected tropical disease
PCR: Polymerase chain reaction
px: Pixel
SiLU: Sigmoid linear unit
TN: True negative
TP: True positive
VL: Visceral leishmaniasis
WHO: World Health Organization
YOLO: you only look once
